# A Novel Approach for Correcting Limb Length Discrepancy in Total Hip Arthroplasty

**DOI:** 10.7759/cureus.56628

**Published:** 2024-03-21

**Authors:** Murat Kezer, Yusuf Onur Kizilay

**Affiliations:** 1 Department of Orthopedics and Traumatology, Private Aritmi Osmangazi Hospital, Bursa, TUR; 2 Department of Orthopedics and Traumatology, Medical Park Hospital, Bursa, TUR

**Keywords:** spirit level, measurement, hip, total hip arthroplasty, limb length discrepancy

## Abstract

Introduction: This study aimed to evaluate the accuracy of the suture technique, along with the utilization of a spirit-level device which is an instrument designed to indicate whether a surface is horizontal or vertical, in comparison to the conventional supraacetabular pin method and caliper measurement for correcting leg length discrepancy (LLD) during total hip arthroplasty (THA).

Materials and methods: Consecutive patients who underwent unilateral primary THA between January 2021 and March 2023 were included in the study. The exclusion criteria were severe flexion and adduction contracture that could affect the accuracy of measurements, a history of lower extremity surgery, the presence of lower extremity deformity, and the absence of postoperative full-length radiographs. Patients were allocated into two groups based on the technique employed for correcting LLD during THA. Group 1 (n=62) consisted of patients evaluated using the suture technique with a spirit-level device, while group 2 (n=75) comprised patients who underwent the supraacetabular pin method with caliper measurement. The distance between the inter-teardrop line and the tip of the lesser trochanter was measured for both hips to assess LLD.

Results: The mean preoperative LLD was similar between groups, which was 11.6 ± 9.1 mm in group 1 and 9.5 ± 9.8 mm in group 2 (p=0.191). Postoperatively, group 1 had a significantly lower LLD compared to group 2 (p<0.001).

Conclusion: According to the results obtained from this study, the use of a suture technique in conjunction with a spirit-level device to achieve a consistent leg position is an effective method for correcting LLD during THA.

## Introduction

Achieving stability and equal leg lengths during total hip arthroplasty (THA) is crucial, not only for providing substantial pain relief but also for restoring optimal hip function [1]. Studies have reported that the occurrence of leg length discrepancy (LLD) after THA can be as high as 62% [2]. LLD can result in a range of complications, including back pain, limping, dislocation, nerve damage, patient dissatisfaction, and an increased likelihood of early revision surgeries. Furthermore, it is one of the primary causes of legal actions against orthopedic surgeons [2-5].

Preoperative and postoperative radiographic assessments, including the use of anteroposterior (AP) and full-length pelvic radiographs, along with templating, have demonstrated their potential to reduce complications associated with THA [6,7]. Despite their utilization, however, the occurrence of LLD may persist [8,9].

Several intraoperative methods exist for measuring and evaluating LLD. One commonly used approach involves the utilization of a fixed reference point anchored to the ilium, such as a Steinman pin or K-wire, coupled with a fixed point located at the greater trochanter [10]. An alternative to this conventional pin measurement method is the suture technique, which offers a simple and non-invasive solution [11]. The accuracy of measurements in these methods is highly dependent on leg positioning, similar to abduction and adduction contracture, impacting limb length measurements and necessitating parallel alignment with the floor for precision [12].

Few studies have previously examined the effectiveness of utilizing spirit-level devices to achieve leg parallelism during leg length measurements in THA [13,14]. Until March 2022, our center employed the conventional supraacetabular pin method and caliper measurement to correct leg length discrepancy based on preoperative radiographs. However, starting in March 2022, we transitioned to using the suture technique alongside a spirit-level device to ensure the parallel alignment of the extremity with the floor. This study aimed to evaluate the accuracy of the suture technique, along with the utilization of a spirit-level device, in comparison to the conventional supraacetabular pin method and caliper measurement for correcting LLD during THA. Our hypothesis suggested that the less invasive suture technique, in conjunction with the spirit-level device, would yield improved accuracy compared to the conventional pin method.

## Materials and methods

Study population

**Figure 1:**
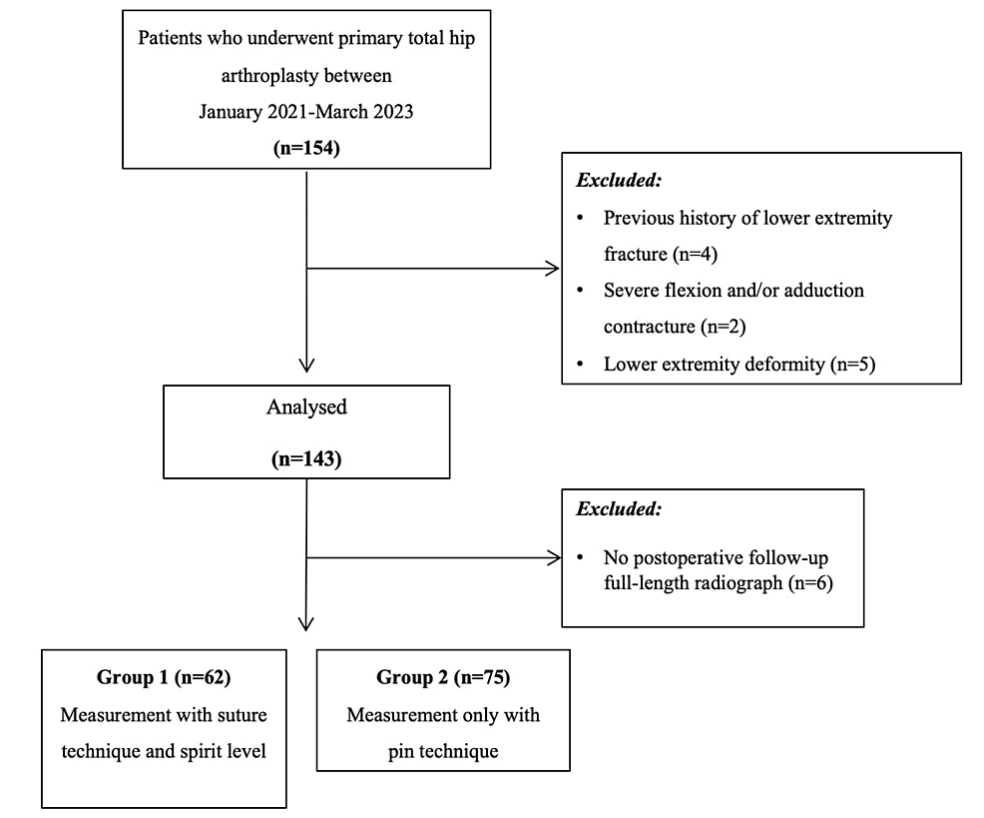
Flowchart diagram of the study

This retrospective comparative study was approved by the Atlas University Clinical Research Ethics Committee (E-22686390-050.99-28135). The study was conducted in accordance with the principles of the Declaration of Helsinki. We retrospectively reviewed patients who underwent primary THA at the Private Aritmi Osmangazi Hospital between January 2021 and March 2023. Consecutive patients who underwent unilateral primary THA within this period were included in the study. Exclusion criteria comprised severe flexion and adduction contracture that could affect the accuracy of measurements, a history of lower extremity surgery, the presence of lower extremity deformity, and the absence of postoperative full-length radiographs. Patients were allocated into two groups based on the technique employed for correcting LLD during THA. Group 1 (n=62) consisted of patients evaluated using the suture technique with a spirit-level device, while group 2 (n=75) comprised patients who underwent the supraacetabular pin method with caliper measurement (Figure 1).

Measurement technique

**Figure 2:**
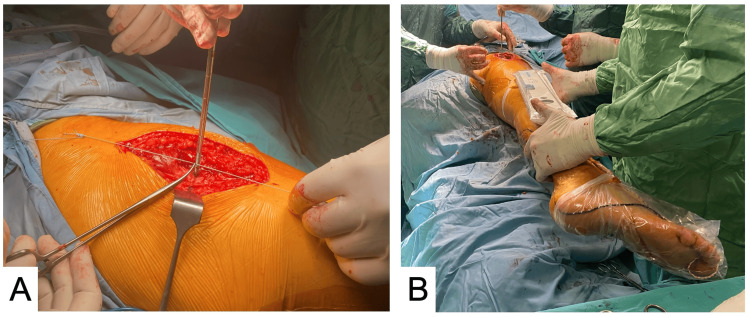
(A) The suture was firmly secured with a clamp. (B) Throughout this process, the leg remained in a parallel position, facilitated by the use of a spirit-level device

All patients in this study were operated on by the same surgeon, the senior author of this study (MK). The surgeries were performed with patients in the lateral decubitus position, utilizing the lateral Hardinge approach. After dissecting the fascia layer and removing the bursa over the greater trochanter, a precise point was marked using an electrocautery device. In group 1, the suture was tied approximately 5 cm proximal to the wound site. To mark the leg of the suture, a surgical marker was utilized at the point on the greater trochanter. Subsequently, the marked suture was firmly secured with a clamp. Throughout this process, utmost care was taken to ensure that the leg remained in a parallel position, facilitated by the use of a spirit-level device (Figure 2).

**Figure 3:**
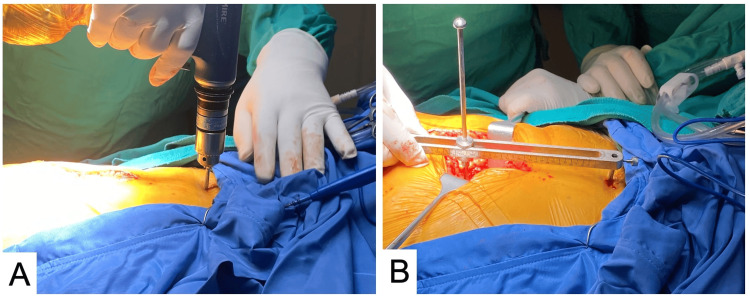
(A) A percutaneous Steinmann pin was sent into the ilium as a reference. (B) The device included a ruler that measured the difference between the two pins to assess LLD LLD: Leg length discrepancy

In group 2, a percutaneous Steinmann pin was inserted into the ilium as a reference, while another pin was placed at the marked point on the greater trochanter. The device included a ruler that measured the difference between the two pins to assess LLD (Figure 3). After the reduction of the hip using the trial femoral stem, neck, and head, the same measurements were subsequently repeated. In instances where a discrepancy was observed, necessary adjustments were made by modifying the vertical offset of the femur, employing different head and femur trial components. A final measurement was conducted subsequent to the implantation of the femoral component and the reduction of the hip joint.

Data evaluation

Radiographic measurements were obtained using preoperative and postoperative pelvis AP radiographs and full-length radiographs. Prior to surgery, the senior surgeon performed all preoperative measurements for surgical planning purposes. Postoperative measurements were also conducted by the same surgeon and documented for all patients. The distance between the inter-teardrop line and the tip of the lesser trochanter was measured for both hips. The lengths of the femur and tibia, along with coronal alignment angles, were measured to exclude lower extremity deformities and concomitant LLD unrelated to the hip joint. Measurements were performed through the PACS software of our hospital.

Statistical analysis

The statistical analysis of the study was conducted using IBM SPSS Statistics for Windows, Version 26 (Released 2019; IBM Corp., Armonk, New York, United States). Numerical variables were presented as means and standard deviations, while categorical data were reported as frequencies and percentages. Student t-test was used for comparing means, and the chi-square test was employed for comparing frequencies. Statistical significance was determined with a threshold of p<0.05.

## Results

Group 1 consisted of 62 patients (40 female, 22 male) with a mean age of 55.4 years (±14.3), and group 2 consisted of 75 patients (47 female, 28 male) with a mean age of 58.9 years (±12.8). Regarding side distribution, 35 (56.4%) of group 1 patients had their condition on the right side and 27 (43.6%) on the left side, while in group 2, these percentages were 44 (58.6%) and 31 (41.4%), respectively. Crowe classifications of the study groups were demonstrated in Table 1. No statistically significant differences were found between the two groups in terms of age, side, and Crowe classification.

**Table 1:** Preoperative clinical characteristics of the patients *: p-value according to Student t-test; **: p-value according to chi-square test; LLD: Leg length discrepancy; n: Number

	Group 1 (n=62)	Group 2 (n=75)	p-value
Age (years)	55.4 (±14.3)	58.9 (±12.8)	0.127*
Gender			0.823**
Female	40 (64.5%)	47 (62.6%)	
Male	22 (35.5%)	28 (37.4%)	
Side			0.794**
Right	35 (56.4%)	44 (58.6%)	
Left	27 (43.6%)	31 (41.4%)	
Crowe classification			0.193**
Crowe 1	39 (62.9%)	58 (77.3%)	
Crowe 2	15 (24.2%)	8 (10.6%)	
Crowe 3	7 (11.2%)	8 (10.6%)	
Crowe 4	1 (1.6%)	1 (1.3%)	

Before surgery, there was no significant difference in leg length difference (LLD) between the two groups, with group 1 having an LLD of 11.6 mm (±9.1) and group 2 having 9.5 mm (±9.8). However, after surgery, a significant difference in LLD values was observed between the groups. In group 1, the LLD was 2.0 mm (±2.5), whereas in group 2, it was 4.6 mm (±5.0) (Table 2).

**Table 2:** Preoperative and postoperative measurements with p-values *: p-values according to the Student t-test, LLD: Leg length discrepancy; n: Number; mm: Millimeter

	Group 1 (n=62)	Group 2 (n=75)	p-value*
LLD	mean (SD) (mm)	mean (SD) (mm)	
Preoperative	11.6 (±9.1)	9.5 (±9.8)	0.191
Postoperative	2.0 (±2.5)	4.6 (±5.0)	<0.001

## Discussion

The most significant finding of this study was the achievement of significantly better LLD correction through the suture technique combined with a spirit level for leg positioning, compared to the conventional pin and caliper measurement. The outcomes of the current study support the acceptance of our null hypothesis, indicating that calibrating the leg position using a spirit-level device led to a more accurate assessment of LLD with the non-invasive suture technique.

Preoperative templating plays a crucial role in determining the position of implants and assessing LLD. Despite pelvic variations, the greater trochanter serves as a common reference point for evaluating leg length both before making the neck cut during trial checks and after implantation. Since the first description of the intraoperative leg length assessment technique in 1985 by McGee and Scott, many techniques have been utilized to equalize leg length during THA [15]. Controlled studies have reported better outcomes with pin and caliper measurements compared to traditional methods [16-18]. In these studies, L-shaped pins are used to achieve a parallel axis to the floor.

Up to a 60% mismatch, as reported in the literature, in preoperative templating has prompted orthopedic surgeons to employ intraoperative techniques. Achieving an accurate leg position is crucial, and the proper alignment of the leg and the surgical table is of utmost importance during intraoperative evaluation [19]. This issue has also led to the development of computer navigation techniques for better measurement accuracy [20]. Chen et al. designed a simple device with a pin and a spirit level to hold the leg in the same position during measurements. The authors reported a 2.5 mm LLD compared to traditional methods, which showed a 6.2 mm difference [13]. Another study compared three methods leg-to-leg comparison, a spirit-level device, and a trochanter/joint ratio device. Nossa et al. mentioned that a >5mm difference was observed in 31%, 27%, and 15% of patients, respectively [14]. To date, we could find only these two studies that used a spirit level to calibrate the leg position during repeated measurements. In our study, we employed a simple suture technique and a commercial spirit-level device and achieved a mean difference of 2.0 ± 2.5 mm (ranges, -10 to 6 mm).

Promising outcomes have been reported in the literature regarding suture techniques for intraoperative LLD assessment. Papadopoulos et al. reported a mean LLD of 1.58 mm (ranges, -8 to 7 mm) in their study using a suture tied proximal to the wound and measuring the distance at a fixed point on the greater trochanter [11]. The suture technique is a minimally invasive method without the necessity to penetrate bone with a pin. However, the skin is not a fixed point like bone, which may affect the measurements due to loosening and tension. It is crucial to maintain consistent tension in the suture.

Desai et al. introduced another method with a suture that is tied to the iliac pin [21]. In the current study, we also maintained consistent tension in the suture while measuring the distance from the greater trochanter point. Nevertheless, leg abduction and adduction may impact the measurements during consecutive assessments. Thus, we employed a spirit-level device located over the lateral cruris and set the bubble levels in the center to ensure the same position and achieve a parallel alignment to the floor. To date, no study has utilized this technique. Sarin et al. also reported changes in measurements related to leg position in their computer navigation study [12].

The main limitation of this study was the absence of a spirit-level device in the conventional pin and caliper measurement groups. Similar outcomes could potentially be achieved if the leg were positioned according to a spirit-level device in the conventional pin method. On the other hand, the results of the current study revealed that LLD can be corrected by a non-invasive suture method, which is augmented with a spirit-level device. This represents the first clinical study in the literature evaluating the correction of LLD using the suture method and a spirit-level device in comparison to the conventional pin and caliper measurement method.

## Conclusions

According to the results obtained from this study, the use of a suture technique in conjunction with a spirit-level device to achieve a consistent leg position is an effective method for correcting LLD during THA.
